# Thermal Stability of Gel Foams Stabilized by Xanthan Gum, Silica Nanoparticles and Surfactants

**DOI:** 10.3390/gels7040179

**Published:** 2021-10-22

**Authors:** Youjie Sheng, Canbin Yan, Yang Li, Yunchuan Peng, Li Ma, Qiuhong Wang

**Affiliations:** College of Safety Science and Engineering, Xi’an University of Science and Technology, Xi’an 710054, China; canbin2021@163.com (C.Y.); liyang5567@163.com (Y.L.); pengyunchuan33@163.com (Y.P.); mal@xust.edu.cn (L.M.); wangqiuhong1025@126.com (Q.W.)

**Keywords:** foam, thermal stability, xanthan gum, nanoparticle, surfactant

## Abstract

The foams stabilized by nanoparticles (NPs), water-soluble polymers, and surfactants have potential application prospects in the development of new, environmentally friendly firefighting foams. In the present study, a gel foam containing a water-soluble polymer (xanthan gum, XG), hydrophilic silica NPs, hydrocarbon surfactant (SDS), and fluorocarbon surfactant (FS-50) were prepared. The surface activity, conductivity, viscosity, and foaming ability of foam dispersions were characterized. The gel foam stability under a radiation heat source and temperature distribution in the vertical foam layer were evaluated systematically. The results show that the addition of NPs and XG has a significant effect on the foaming ability, viscosity and foam thermal stability, but has a very subtle effect on the conductivity and surface activity. The foaming ability of the FS-50/SDS solution was enhanced by the addition of NPs, but decreased with increasing the XG concentration. The thermal stability of the foams stabilized by SDS/FS-50/NPs/XG increased with the addition of NPs and increasing XG concentration. Foam drainage and coarsening were significantly decelerated by the addition of NPs and XG. The slower foam drainage and coarsening are the main reason for the intensified foam thermal stability. The results obtained from this study can provide guidance for developing new firefighting foams.

## 1. Introduction

Foam is the assemblage of gas bubbles dispersed in a continuous liquid phase, where the gas phase may be of air or other gas, and the liquid phase consists of mostly surfactant solutions. Fundamental studies of foam have gained profound interest over the years because of their vast applications in various industries, such as food [1], froth floatation [2,3], cosmetics [4], firefighting [5,6] and petroleum industries [7]. In the process of oil extraction, transportation, storage and use, the occurrence of oil fire is inevitable. Aqueous film-forming foams (AFFFs) are considered the most effective fire extinguishing agent in fighting liquid fuel fires. The critical components of AFFF are hydrocarbon surfactants and fluorocarbon surfactants. Hydrocarbon surfactants are good foaming agents, and fluorocarbon surfactants are used to improve the surface activity of AFFF solutions [8], which are very important for AFFF to fight liquid fires. However, the use of traditional long-chain fluorocarbon surfactants (C8–C10) has been totally restricted by the United Nations Environment Program because of their severe ecological hazards [9,10,11]. The new environmentally friendly foam fire extinguishing agent currently researched shows poor fire extinguishing and burn-back performance [12,13,14]. Performance-enhanced foam stabilizers are worth studying.

Theoretically, foam is thermodynamically and kinetically unstable, and faster collapse makes it unfavorable for many practical and industrial applications. Water-soluble polymers, such as xanthan gum and carboxymethyl cellulose, are excellent foam stabilizers [15,16]. The polymer and surfactant mixture in the solutions has been thoroughly studied for its major applications in such industries as food, cosmetics, and oil [1,4,17]. The mixture of surfactants and polymers in the solutions can give rise to molecular interactions, thereby affecting the properties of the solution, such as the surface tension, viscosity, and conductivity [18,19]. Polymers can enhance aqueous solution viscosity and the corresponding foam stability, so they can be added as a highly effective foam-stabilizing agent in AFFF concentrates [18]. Polymer- and surfactant-stabilized foams collapse and evaporate quickly at high temperatures because there is no stable support between the bubbles. So, the major problem of using the foam in the fire extinguishing job is its thermal stability.

Previous studies have shown that nanoparticles (NPs) are able to improve the foam stability of surfactants, including the NPs of SiO_2_ [5,6,7], CaCO_3_ [20], Al(OH)_3_ [21], and so forth. The NPs provided a high stability for surfactant foams by decelerating foam drainage and delaying foam coarsening between bubbles [22,23,24,25], due to their aggregates in bubble films and plateau borders. The factors affecting the ability of NPs stabilizing foam were investigated, such as types, size, and concentrations of NPs [5,20,21,26]. Researchers have conducted in-depth studies on the foam stabilization mechanism of nanoparticles and surfactant dispersions, foaming ability, foam stability, etc. [27,28]. However, the previous studies were mainly focused on individual surfactants and their mixtures with nanoparticles or water-soluble polymers [17,29,30,31]. The addition of polymers and nanoparticles to a surfactant system can further help to improve the foam stability [32]. However, very limited focus has been given to the interaction between nanoparticles, polymers and the surfactant system for improving foam stability [33,34,35]. Abro et al. reported the addition of xanthan gum (XG) to carbon foam for its heat absorption and retardation properties [36]. However, it is unclear whether XG can improve the thermal stability of nanoparticle-stabilized firefighting foams.

In the present study, a gel foam stabilized by NPs, xanthan gum, and surfactants was prepared. The synergistic effect of xanthan gum and NPs to improve the gel foam thermal stability was analyzed systematically. The viscosity, conductivity, surface tension and foaming ability of the mixed dispersions of the gel foams were investigated. The volume change and internal temperature distribution of the gel foams under different temperature were studied and compared with the traditional gas–liquid two-phase foam. The results obtained from this study provide a basis for the application of the new prepared gel foams in firefighting.

## 2. Results and Discussion

Table 1 shows the formulations of the gel foam dispersions, consisting of sodium dodecyl sulfate (SDS), amphoteric fluorocarbon surfactant (FS-50), NaCl, XG, NPs, and deionized water.

### 2.1. Properties of Foam Dispersions

**Figure 1:**
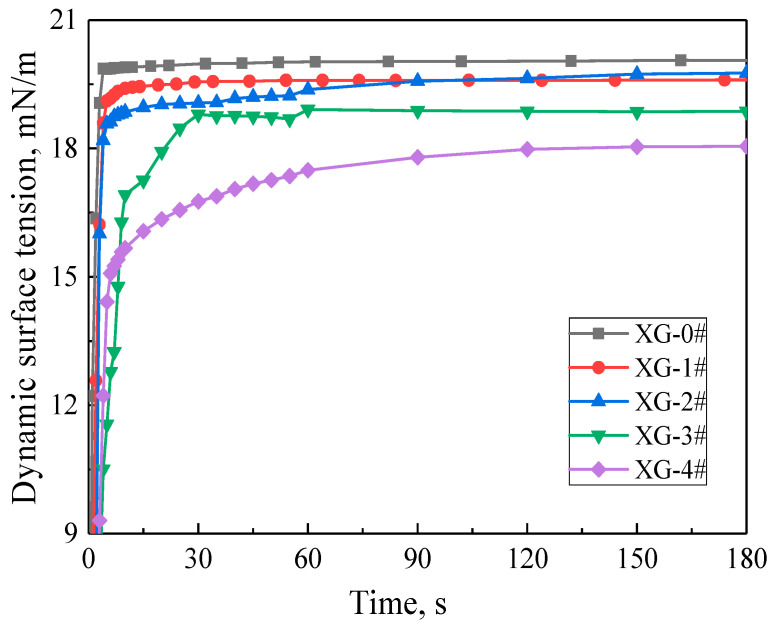
Dynamic surface tension of the foam dispersions.

The dynamic surface tension (DSTs) of each foam dispersion changes with time, as shown in Figure 1. The platinum flakes touching the surface of the dispersion and the surface tension value increases sharply from zero in only a few seconds. Then, the surface tension slowly increases with time and reaches a relatively stable value after a brief increase. The stable value is called the equilibrium surface tension (EST). The surface tension of the foam dispersion (XG-0#, XG-1#) without XG tends to balance at 30 s. The addition of XG delays the time for the dispersion to reach EST, and the higher the concentration of XG, the longer it takes to reach EST, but all the foam dispersions reach EST within 150 s. The ESTs of XG-1# and XG-2# dispersions are almost equal, but the DST curve of XG-2# is lower than that of XG-1# before reaching the EST. In addition, the DST curve gradually decreases, indicating that the surface activity gradually increases as the XG concentration increases. The EST value of XG-0# is 20.041 mN/m, which is between the EST of individual FS-50 solution at cmc (13.89 mN/m [28]) and the EST of individual SDS solution at cmc (34.43 mN/m [30]). The results indicate that SDS and FS-50 molecules coexist at the gas–liquid interface. The EST of XG-4# is 18.094 mN/m, indicating that the addition of NPs and XG enhanced the surface activity of SDS and FS-50.

The EST, conductivity and dynamic viscosity of the foam dispersion are shown in Table 2. For the SDS/FS-50 dispersion containing NP without XG, the surface tension and conductivity decreased slightly, and the viscosity increased slightly. Adding 0.01% XG to the dispersion of SDS/FS-50/NPs resulted in a slight increase in conductivity, but the conductivity gradually decreased with the increase in the XG concentration. The dynamic viscosity of the SDS/FS-50/NPs mixture increased sharply with the increase in XG concentration. In particular, at the concentration of 5% NPs and 0.05% XG, the dynamic viscosity of foam dispersions reached 870.58 mPa‧s, implying that the molecular interactions among surfactants, XG, and NPs resulted in a synergistic thickening effect. The main reason is that the relative molecular of XG mass exceeds a million, and it is known to be highly entangled in solution. The macromolecular dimensions and molecular weight of XG increased effectively because of the intermolecular interaction or intense entanglement of the XG molecules in solution. The effectively increased macromolecular dimensions and molecular weight improved the viscosity of the mixed solutions.

**Figure 2:**
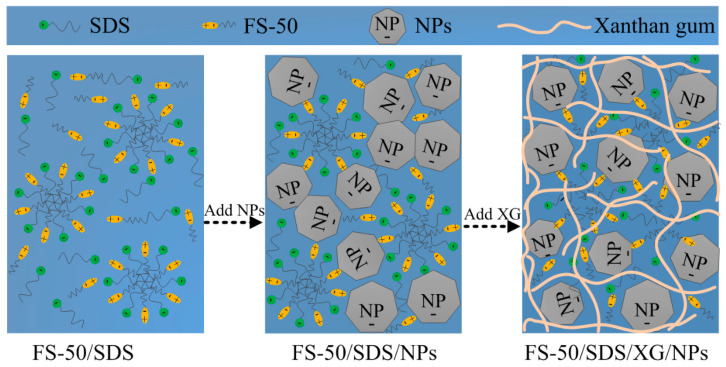
Schematic diagram of the molecular interaction in bulk dispersions.

The difference between the EST values of the foam dispersions was only about 2 mN/m with the increasing concentration of XG and NPs, indicating that XG and NPs had a slight effect on the molecular interactions of FS-50/SDS at the air/liquid interface. However, the conductivity and viscosity of the dispersions were affected significantly by the intense molecular interactions between the molecules of FS-50/SDS/NPs/XG in the bulk solutions. The schematic diagram of the molecular interaction in the mixed dispersions of FS-50/SDS/NPs/XG is shown in Figure 2.

XG is an anionic polymer, which is negatively charged in water [37]. Silica NPs are also negatively charged in water [38]. SDS is anionic, and FS-50 is zwitterionic. Hence, Coulomb forces existed in the same charged molecules of FS-50/SDS/NPs/XG [39]. In addition, opposite attraction existed between the zwitterionic FS-50 and the negatively charged SDS/NPs/XG. The complicated and intense molecular interactions led to the change in conductivity and viscosity. This result showed that XG stabilized stronger molecular interactions with SDS/FS-50/NPs dispersions than with NPs alone.

### 2.2. Foaming Ability

**Figure 3:**
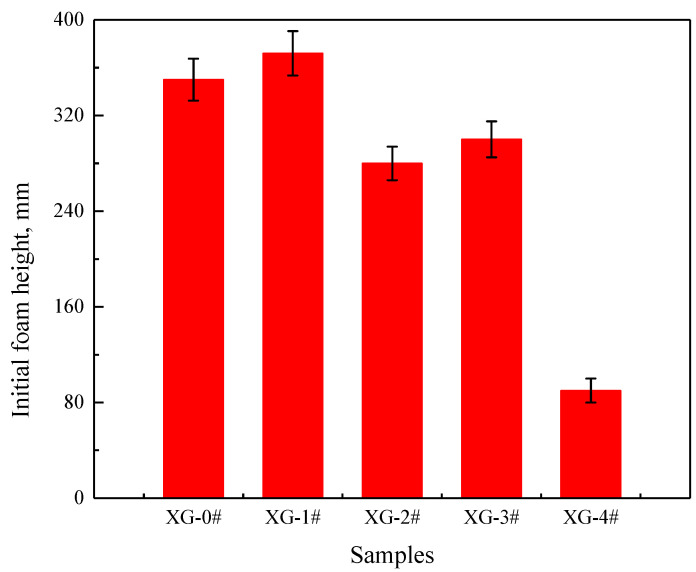
Foaming ability of the foam dispersions.

Figure 3 shows the foaming ability of the five dispersions. The addition of 5% NPs into the SDS/FS-50 solution led to the enhancement of the foaming ability. However, the addition of 0.01% XG into the SDS/FS-50/NPs dispersion reduced the foaming ability, while the foaming ability of SDS/FS-50/NPs dispersion increased slightly, as the XG concentration was 0.03%. However, the foaming ability of SDS/FS-50/NPs dispersion decreased dramatically at the concentration of 0.05% XG. Previous studies have shown that the increase in viscosity hampered bubble formation and the foaming ability would be significantly reduced [30]. The reason for the changes in the foaming ability in the present study should be the complicated molecular interactions and the dramatic increase in viscosity.

### 2.3. Foam Thermal Stability

#### 2.3.1. Foam Drainage and Decay Process

**Figure 4:**
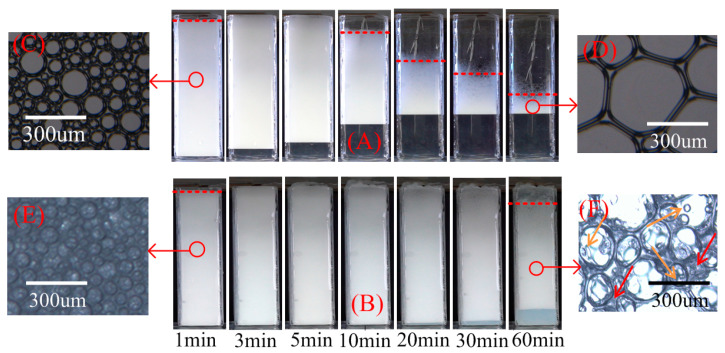
Process of foam decay and drainage under T = 200 °C: (**A**) XG-0#, (**B**) XG-4#, (**C**,**E**) foam morphology at zero, (**D**,**F**) foam morphology at 60 min.

Figure 4 shows the foam decay process under 200 °C radiation heat source (only XG-0# and XG-4# for convenience). Generally, the foam volume decreases gradually after a slight expansion. Foam expansion occurred as the gas in the foam bubbles was heated continuously and then stabilized for a period of time. As time went on, the foam began to collapse due to the rapid evaporation and drainage of the liquid in the foams. The time required for XG-0# and XG-4# foam volume to expand to the maximum values was 5 min and 10 min, respectively. The foam volume expansion of XG-4# was more obvious and the time of the foam expansion stage lasted longer than that of XG-0#. Almost all the liquid of XG-0# foam drained out within 30 min, while the liquid drained out of the XG-4# foam was still very small, even at 60 min. After 60 min, the XG-0# foam was almost completely ablated, but there was still a large amount of foam in XG-4#, and the appearance of the XG-4# foam did not change significantly. The foam decay process indicated that the presence of NPs largely enhanced the foam thermal stability.

The initial foam morphology of the dispersion and the morphology of the foam after 60 min were observed under a microscope, as shown in Figure 4C–F. It can be seen from the initial shape of the foam that when the foam was observed under a microscope, XG-0# had many two-dimensional bubbles, while XG-4# bubbles were all three-dimensional spherical structures. The size of the bubbles increased gradually over time, and the shape of the bubbles changed from a three-dimensional sphere to a two-dimensional polygon, and the number of bubbles was reduced dramatically because of foam coarsening caused by the pressure difference among bubbles with different sizes [40]. However, for foams containing XGs and NPs, the bubbles were irregular in shape; the thickness and radian of the bubble films were uneven, and a gel-like membrane occurred in the films. The formation of a gel-like membrane was attributed to the addition of XG [19]. XG-4# bubbles maintained their three-dimensional structure, and there were obvious gel-like films at the apex and bubble film. A large number of small bubbles with several tens of microns in diameter remained in the XG-4# image at 60 min (orange arrows). Furthermore, a large number of shadows covered the films, vertices, and bubbles of XG-4# (red arrows). The shadows were due to the aggregates of NPs, XG, and surfactant molecules. Foam coarsening was delayed or even stopped by aggregates and XG. Apparently, the foam containing XG and NPs is more stable.

#### 2.3.2. Variation in Foam Thickness and Drainage Height Versus Time

**Figure 5:**
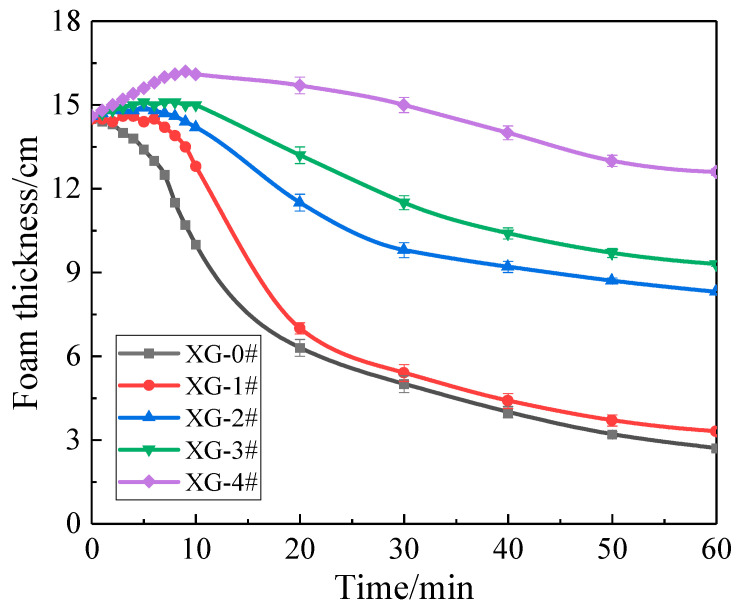
Variation in foam thickness under 200 °C versus time.

To quantitatively analyze the foam decay process under radiation heat, the foam thickness (the height of upper surface of foam layer minus the height of the interface between the foam layer and the liquid drained) change versus time was measured, as shown in Figure 5. The foam thickness curves gradually increased with the increasing XG concentration. In particular, the foam thickness curves of XG-1#, XG-2#, XG-3#, and XG-4# gradually increased after the heating started, and then gradually decreased over time. It should be ascribed to foam expansion under heat at an early stage, as described in Figure 4. The maximum foam thickness of XG-1#, XG-2#, XG-3#, and XG-4# were 14.6, 14.9, 15.1, and 16.2 cm, reached at 3, 5, 7, and 9 min, respectively. After a brief stabilization near the peak, the bubble began to collapse. However, the foam thickness curve of XG-0# kept decreasing from the beginning, which is inconsistent with the phenomenon of the foam expansion stage in Figure 4. The results show that the increase in XG concentration led to the enhancement of the foam layer stability under heat. 

The main reason is the rapid foam drainage. The foam drainage height is greater than that of foam expansion for XG-0#, resulting in the continuous decrease in the foam thickness (the difference between foam expansion height and foam drainage height). Hydrophilic NPs would aggregate and form a network structure in the foam films and plateau borders, thereby stopping foam drainage [40]. The addition of XG made the Coulomb repulsive interaction between the same charged molecules of SDS/NPs/XG strengthen the arrangement of NPs in the foam film and platform boundary. This network structure makes the foam layer have a stable skeleton, as the upper layer of foam is evaporated under heat radiation to leave a non-combustible nanoparticle layer, and the lower layer of foam is protected. Therefore, NPs and XG-stabilized foams decay more slowly, and the foam layer stability under heat is enhanced.

**Figure 6:**
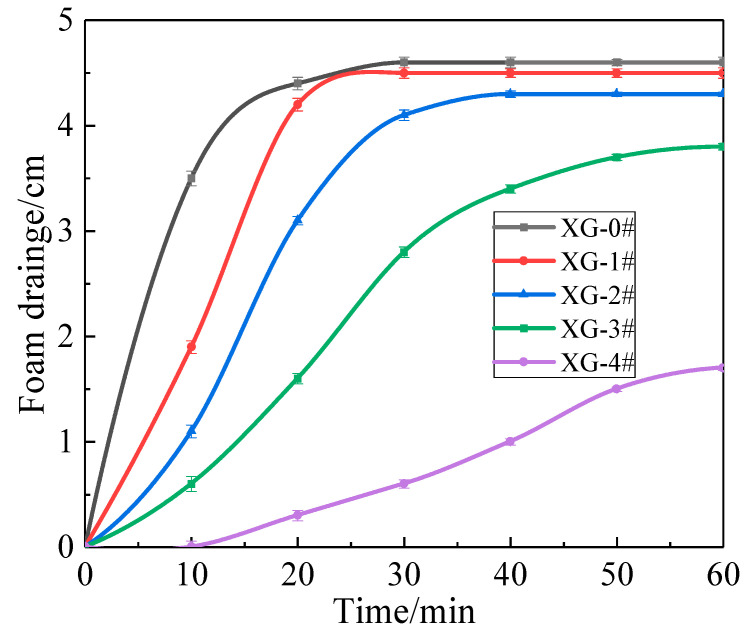
Variation in foam drainage height under 200 °C versus time.

Figure 6 shows the height of liquid drained out of foams under thermal radiation. The drainage height of all foams increased over time. The foam drainage height curves gradually declined from XG-0# to XG-4#, implying that foam drainage was decelerated with increasing XG concentration. The maximum drainage heights were 4.6, 4.5, 4.3, 3.8, and 1.7 cm, respectively. Notably, XG-4# began to drain slowly after it was heated 10 min, and the final foam drainage height was only 1.7 cm after 60 min. The results show that the foam containing NPs is more stable than the gas–liquid two-phase foam. The addition of NPs and XG further enhanced the thermal stability of the gel foam.

#### 2.3.3. Temperature Distribution of the Foam Layer

**Figure 7:**
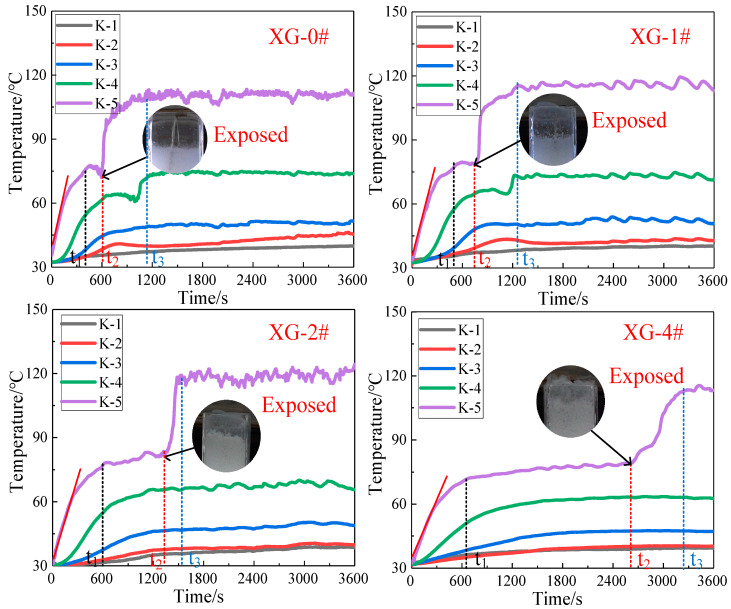
Temperature distribution inside foam layer at different positions under 200 °C.

The temperature at different depths inside the foam layer is shown in Figure 7. K-1–K-5 represent the temperature change curve of thermocouples at different depths in the foam layer. Generally, all the temperature curves increased over time. For all the experiments, the temperature curves of K-1, K-2, and K-3 exhibited a similar change trend, that is, they increased slowly over time, and reached a stable temperature value after 1200 s. However, the temperature curves of K-4 and K-5 showed different trends. For XG-2# and XG-4#, the curves of K-4 showed the same trends as those of K-1, K-2, and K-3. However, K-5 of all four experiments and K-4 of XG-0# and XG-1# exhibited ladder-shaped curves. The ladder-shaped curve can be explained by whether the thermocouples were exposed to air or not. At the beginning of the experiment, the temperature of the foam layer increased gradually over the heating time. The temperature values of the foam layer around K-5 reached approximately 80 °C at t1 and kept relatively stable. At this period, K-5 was still wrapped in the foam layer. The temperature around K-5 showed the second rapid increase at t2. The reason is that the foam around K-5 disappeared completely under heat at t2, and K5 was directly exposed to the heat source. The K-5 curves reached a relatively stable temperature value again at t3. For K-4 curves of XG-0# and XG-1#, the thermocouples of K4 were also directly exposed to air under the heat source at a moment close to t3.

It is the maximum value that K-5 reached in air under the heat source used in the present study. The curves of K-1, K-2 and K-3 are relatively smooth. Meanwhile, the K-4 and K-5 curves show fluctuation changes after t3. The main reason is that the thermocouples were completely buried in the foam. The surrounding temperature is relatively stable, and the corresponding temperature values obtained from the thermocouples are relatively stable. When the thermocouples were exposed to the air, the temperature fluctuated slightly, due to air flow. Apparently, greater t1, t2, and t3 means the stronger ability for foam to resist heat radiation. In addition, for the same position in different experiments, the smaller the temperature values, the stronger the ability for foam to resist heat radiation.

The time, t1, t2, and t3, and the temperature values (Tt1 and Tt3) reached at t1 and t3 are listed in Table 3. In addition, a linear fitting was performed on the K5 curves before t1, and the slope was recorded as K(0–t1), also listed in Table 3. All the values of t1, t2, and t3 increased gradually from XG-0# to XG-4#. The values of Tt1 and Tt3 were almost unchanged from XG-0# to XG-4#, but the time at which the temperature was kept at the relatively stable value of Tt1 increased obviously. The values of K(0–t1) decreased gradually. These results indicate that it is more and more difficult for heat to transfer through foam layer with the addition of NP and increasing XG concentration. In other words, the thermal insulation performance of the gel foams gradually increased with the addition of NP and increasing XG concentration. Hence, we can conclude that the addition of NPs resulted in enhancement of the thermal stability of foams stabilized by SDS/FS-50 surfactant solutions. The addition of XG led to further enhancement of the thermal stability of foams stabilized by SDS/FS-50/NPs dispersions. Additionally, the thermal stability of foams stabilized by SDS/FS-50/NPs/XG kept increasing with increasing XG concentration.

The NP layer formed in the bubble films and plateau borders can hinder heat transfer to some extent and also contribute to thermal insulation [41,42]. In the present study, the main reason for the enhancement of foam thermal stability should be ascribed to the significant deceleration of foam drainage and coarsening caused by the addition of NPs and XG. For the slower drainage of foam, a large amount of liquid remained in the foams, resulting in more heat absorbed by the foam layer through evaporation before foam collapse. The slower foam coarsening resulted in poorer gas diffusion among bubbles, which can effectively prevent heat transfer from the upper heated gas in bubbles to the lower bubbles.

## 3. Conclusions

In the present study, a gel foam consisting of water-soluble polymer XG, nanoparticles, and hydrocarbon and fluorocarbon surfactants was prepared. The surface activity, conductivity and viscosity of the foam dispersions were characterized. The foaming ability and bubble morphology of the gel foams were observed. The thermal stability characteristics of the gel foams were evaluated and compared. The main conclusions are as follows:

The addition of NPs and XG has little effect on the conductivity and surface tension of the FS-50/SDS solution, slightly decreasing with the addition of NPs and increasing XG concentration. The viscosity of the gel foam dispersions increased dramatically with the addition of NPs and increasing XG concentration. The foaming ability of the FS-50/SDS solution was enhanced by the addition of NPs, but decreased with increasing XG concentration.

The addition of NPs resulted in enhancement of the thermal stability of foams stabilized by SDS/FS-50 surfactant solutions. The addition of XG led to further enhancement of the thermal stability of foams stabilized by SDS/FS-50/NPs dispersions. In addition, the thermal stability of foams stabilized by SDS/FS-50/NPs/XG kept increasing with increasing XG concentration.

NPs aggregated in bubble films and plateau borders. Foam drainage and the coarsening process were significantly decelerated by NP aggregates, XG, and surfactant molecules among bubble films and plateau borders. The slower foam drainage and coarsening are the main reasons for the intensified foam thermal stability. In addition, by controlling the ratio of XG and NP concentrations, the foaming ability and foam thermal stability can be simultaneously improved.

## 4. Experimental

### 4.1. Materials 

**Figure 8:**
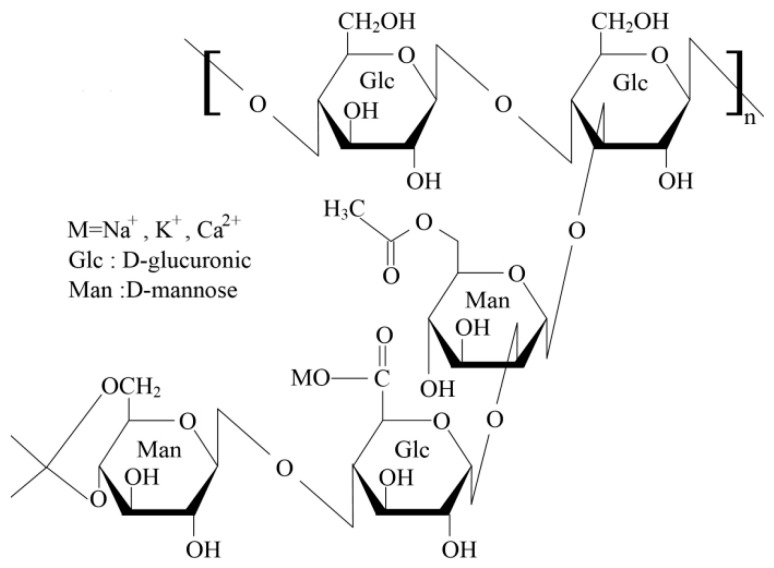
Molecular structures of xanthan gum (XG).

Xanthan gum (XG, 99.9%) was purchased from Tianjin Bailunsi Biotechnology Co., Ltd. Sodium dodecyl sulfate (SDS, 99%) was purchased from Sinopharm Chemical Reagent Co., Ltd. (Shanghai, China). The amphoteric fluorocarbon surfactant, Capstone^®^ FS-50 (C6 in its perfluorinated radical form, 27%), was purchased from Dupont. Figure 8 shown the molecular structural formula of XG [16]. The molecular formula of FS-50 is shown in [38]. The hydrophilic silica NPs (99.8% purity), with a particle size of 7–40 nm and a Brunauer−Emmett−Teller surface area of 300 m^2^/g, were prepared by Shanghai Aladdin Biochemical Technology Co., Ltd. (Shanghai, China).

**Figure 9:**
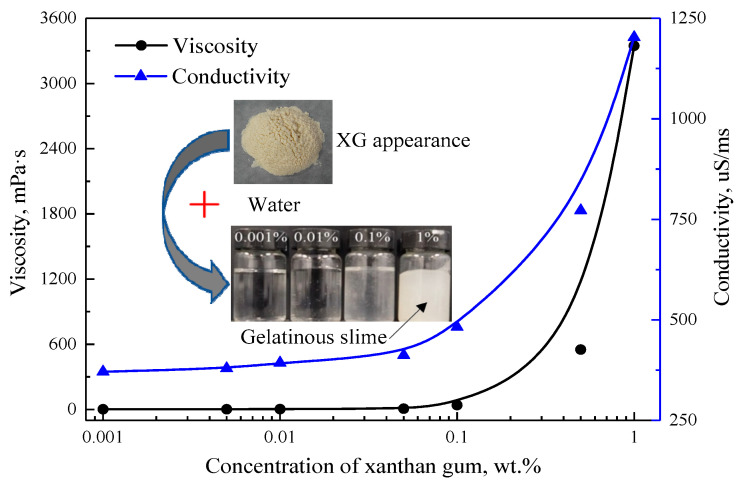
Parameter characterization of XG.

The appearance of XG and its solution’s apparent morphology, viscosity and conductivity changes with concentration are shown in Figure 9. The results show that the viscosity and conductivity of the solution increase sharply, and the solution looks like gelatinous mucus when the concentration of XG exceeds 0.1%, which was unsuitable for the present study. Hence, the low concentrations of 0.01%, 0.03% and 0.05% of XG were selected in this study.

### 4.2. Preparation of Foam Dispersions 

Based on the critical micelle concentration (cmc), the concentrations of SDS and FS-50 were fixed at 16 mM and 0.25 wt%, higher than their cmc of SDS (7.9 mM) and FS-50 (0.0126%) [39,43]. The concentration of NPs was fixed at 5%. The 1 mM NaCl was added to promote the adsorption of surfactant molecules. The foam solution without NPs and XG was numbered as XG-0#. The concentration of XG was 0%, 0.01%, 0.03%, and 0.05%, and the corresponding numbers of the gel foam dispersions were XG-1#, XG-2#, XG-3# and XG-4#, respectively. SDS, FS-50, NaCl, and XG were successively dissolved in 1,000 mL of DI water under magnetic stirring at 800 rpm. Then, 5wt% NPs was added under magnetic stirring at 1500 rpm and sonicated for 20 min (a Scientz-2400F).

### 4.3. Characterization of Foam Dispersions 

**Foam dispersions properties.** The dynamic surface tension of the foam dispersions was measured, using a QBZY-3 fully automatic surface tension meter (platinum plate method). The dynamic viscosity of the foam dispersions was tested, using a DV-1 digital viscometer. The conductivity of the foam dispersions was evaluated by using a SG23-B Mettler multi-parameter tester. In the present study, each experiment was repeated at least three times.

**Foaming ability.** The Ross-Miles method is the standard method for testing foaming ability [44,45]. In this study, the improved Ross-Miles method was used to characterize the foaming ability; the initial foam height of the dispersion represents the foaming ability [46]. The detailed test procedure can be found in Ref. [5].

### 4.4. Testing Device for Foam Thermal Stability

**Figure 10:**
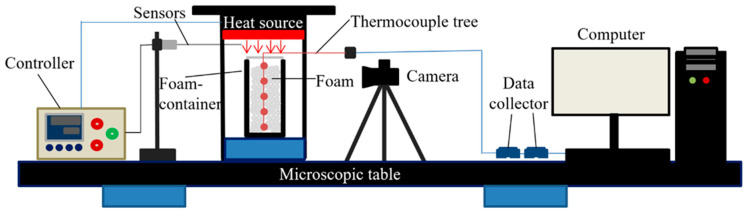
Experimental device for foam thermal stability.

The thermal insulation performance of the foam layer under radiant heat source was studied, using a testing device for foam thermal stability, as shown in Figure 10. A similar experimental setup can be seen in the paper [41,42]. In this study, the device consisted of a set of a SGM series simulated fire field radiation heat source system, sample loading module, and foam container. The radiant heat source system consisted of heating plates, temperature control instruments, sensors, and a stainless-steel bracket. The total heating area of the heating plate was 150 mm × 150 mm, and the heating temperature was adjusted by a temperature control instrument, and the temperature range was 0 ~ 400 °C . The data acquisition module consisted of five K-type armored thermocouples and two data collectors. The foam container was a square quartz glass tube of 2 cm × 3 cm × 15 cm.

During the experiment, the radiant heat source was turned on to preheat to the specified temperature, and five K-type armored thermocouples were arranged in the square tube at intervals of 3 cm in depth. Their position from top to bottom were K5, K4, K3, K2, and K1. The distance between K5 and the lower surface of heat source was 3 cm. A total of 90 mL of foam was added to the square tube and placed under thermal radiation; the temperature data were collected with a computer, and the volume change of foam was recorded with a camera. The foams were prepared by double-syringe foaming technology; the specific operation steps can be seen in Ref. [5]. The foams were generated with the same initial volume for different dispersions based on this method. The bubble distributions are highly reproducible [47]. For each experiment, the volumes of liquid and gas used to generate foam were 30 and 60 mL, respectively.

## Figures and Tables

**Table 1:** Components of foam dispersions.

Samples	SDS (mM)	FS-50 (wt%)	NaCl (mM)	XG (wt%)	NPs (wt%)
XG-0#	16	0.25	1	0	0
XG-1#	16	0.25	1	0	5
XG-2#	16	0.25	1	0.01	5
XG-3#	16	0.25	1	0.03	5
XG-4#	16	0.25	1	0.05	5

**Table 2:** Basic properties of foam dispersions.

Sample	EST (mN/m)	Conductivity (uS/ms)	Viscosity (mPa‧s)
XG-0#	20.041	211	1.22
XG-1#	19.601	202	2.32
XG-2#	19.744	206	53.44
XG-3#	18.867	203.3	370.33
XG-4#	18.094	196.9	870.58

**Table 3:** The values of t1, t2, t3, Tt1, Tt3 and K(0–t1).

Sample	t1 (s)	t2 (s)	t3 (s)	Tt1 (°C)	Tt3 (°C)	K (0–t1)
XG-0#	445	595	1150	78.3	118.5	0.140
XG-1#	518	771	1270	78.5	118.4	0.119
XG-2#	660	1340	1557	78.3	118	0.111
XG-3#	816	1710	2040	76.3	117.1	0.106
XG-4#	805	2675	3270	72.8	116.9	0.087

## Data Availability

Data are contained within the article.
